# Profile of presentation of HIV-positive patients to an emergency department in Johannesburg, South Africa

**DOI:** 10.4102/sajhivmed.v22i1.1177

**Published:** 2021-01-29

**Authors:** Abdullah E. Laher, Willem D.F. Venter, Guy A. Richards, Fathima Paruk

**Affiliations:** 1 Department of Emergency Medicine, Faculty of Health Sciences, University of the Witwatersrand, Johannesburg, South Africa; 2 Ezintsha, Faculty of Health Sciences, University of the Witwatersrand, Johannesburg, South Africa; 3 Department of Critical Care, Faculty of Health Sciences, University of the Witwatersrand, Johannesburg, South Africa; 4 Department of Critical Care, University of Pretoria, Pretoria, South Africa

**Keywords:** HIV, emergency department, ART non-adherence, CD_4_ cell count, HIV viral load, opportunistic infections, hospital admission, mortality

## Abstract

**Background:**

Despite improved availability and better access to antiretroviral therapy (ART), approximately 36% of human immunodeficiency virus (HIV)-positive South Africans are still not virally suppressed.

**Objective:**

The aim of this study was to describe the patterns of presentation of HIV-positive patients to a major central hospital emergency department (ED).

**Methods:**

In this prospectively designed study, consecutive HIV-positive patients presenting to the Charlotte Maxeke Johannesburg Academic Hospital (CMJAH) adult ED were enrolled between 07 July 2017 and 18 October 2018.

**Results:**

A total of 1224 participants were enrolled. Human immunodeficiency virus was newly diagnosed in 212 (17.3%) patients, 761 (75.2%) were on ART, 245 (32.2%) reported ART non-adherence, 276 (22.5%) had bacterial pneumonia, 244 (19.9%) had tuberculosis (TB), 86 (7.0%) had gastroenteritis, 205 (16.7%) required intensive care unit admission, 381 (31.1%) were admitted for ≥ 7 days and 166 (13.6%) died. With regard to laboratory parameters, CD_4_ cell count was < 100 cell/mm^3^ in 527 (47.6%) patients, the viral load (VL) was > 1000 copies/mL in 619 (59.0%), haemoglobin was < 11 g/dL in 636 (56.3%), creatinine was > 120 µmol/L in 294 (29.3%), lactate was > 2 mmol/L in 470 (42.0%) and albumin was < 35 g/L in 633 (60.8%).

**Conclusion:**

Human immunodeficiency virus-positive patients presenting to the CMJAH ED demonstrated a high prevalence of opportunistic infections, required a prolonged hospital stay and had high mortality rates. There is a need to improve the quality of ART services and accessibility to care.

## Introduction

Human immunodeficiency virus (HIV) infection is an epidemic, which has affected approximately 38 million people worldwide. In 2019, 1.7 million new infections and 690 000 HIV-related deaths were recorded.^1^ Two-thirds of the global population of persons living with HIV (PLWH) are in sub-Saharan Africa (SSA). South Africa (SA) contributes approximately 7.5 million to the global number, that is, more than twice that of any other country worldwide.^2^

The availability of antiretroviral therapy (ART) globally has reduced HIV-associated morbidity and mortality rates.^3,4^ Indeed, the life expectancy of PLWH in some regions is now comparable to that of the general population.^4,5^ Although there has been a significant increase in the global number of PLWH on ART in recent years,^6^ the burden of HIV-related illness is still substantial,^7^ especially amongst those who are newly diagnosed, ART-naive and those who were recently initiated on ART.^8^ Other factors contributing to poor HIV-related outcomes include non-adherence to ART, treatment resistance and severe immune deficiency (low CD_4_ cell counts) at the time of presentation.^9^ Furthermore, loss to follow-up (LTFU) remains a problem despite growth in the numbers starting on ART.^4,10^

Emergency departments (EDs) are frequently the first ‘port-of-call’ for PLWH who experience an acute deterioration in health. Despite free access to ART in SA’s public health system,^11^ recent data confirm that approximately 30% of eligible persons are not yet on ART, and of those on ART, 36% are not virally suppressed.^1^ Although these figures have improved since the inception of the ART roll-out in 2004,^12^ admission with acute HIV-related illness is still high. These figures fall short of the 2020/30 Joint United Nations Programme on HIV and AIDS (UNAIDS) 90-90-90 targets.^13^ The country’s healthcare service and in particular it’s EDs depict an early barometer of progress in achieving these goals.

In this study, we describe the profile of presentation of acutely ill PLWH to the ED of a large tertiary hospital. We also describe demographic characteristics, HIV-related history, vital signs, routine laboratory parameters, presenting diagnosis, patient disposition and outcomes.

## Methods

This study was conducted in the adult medical-ED of the Charlotte Maxeke Johannesburg Academic Hospital (CMJAH), a 1088-bed tertiary-level academic hospital affiliated to the University of the Witwatersrand. The adult medical ED manages all non-trauma patients who, on arrival, are triaged as ‘emergent’ (red), ‘very urgent’ (orange), ‘urgent’ (yellow) and ‘routine’ (green) based on specific criteria as defined by the SA Triage Scale.^14^ In general, patients who are triaged as ‘routine’ (green) are referred to a lower-level facility for further management. In addition, patients not residing within the drainage area of the CMJAH, and who are transportation stable, are referred to an appropriate facility closer to the patient’s residence.

Before the commencement of data collection, informal training pertaining to the methodology and principles of data collection from medical charts was undertaken by the primary investigator. Furthermore, all doctors employed in the ED were briefed regarding the aims, objectives and design of the study. Doctors were thereafter requested to inform the primary investigator of all HIV-positive patients managed in the ED. Written informed consent for study participation was obtained by the primary investigator or the doctor on shift. If participants were unable to grant consent (e.g. decreased level of consciousness), consent was obtained from the next of kin/legal guardian and later re-obtained from the participant after his or her mental condition had improved. Human immunodeficiency virus-negative patients, HIV-unknown patients not consenting to HIV testing and patients not consenting to participate in the study were excluded from the study. Emergency department registers were also reviewed daily in an effort to identify potential participants who were missed by the ED doctors.

The four-question AIDS Clinical Trials Group Adherence Questionnaire (ACTG-AQ) was used in order to determine non-adherence to ART.^15^ The questionnaire was administered to all participants prescribed ART at any time in the past.

Data were extracted from the patient’s hospital records by the primary investigator and entered into an anonymised and standardised data collection form. Additional information relevant to the study but not found in the patient’s hospital records was directly obtained from the participant, the participant’s laboratory records or the participant’s next of kin/legal guardian where applicable. Only where the next of kin/legal guardian indicated that they were aware of the participant’s HIV status, they were questioned regarding relevant HIV-history such as treatment adherence. Data from hospital records were recorded daily for the entire duration of hospital stay until data collection was completed. Inter-rater reliability was assessed by an independent researcher experienced in the methods of data collection and blinded to the study aims and objectives. Data extracted from a random sample of 43 medical charts were compared with those extracted by the primary investigator.

Data relevant to this study included demographic details, HIV status, prior ART history including non-adherence, vital signs including the Glasgow Coma Scale (GCS) score, respiratory rate, systolic blood pressure, oxygen saturation, heart rate and temperature, baseline laboratory findings at the time of the current presentation including CD_4_ cell count, HIV viral load (VL), haemoglobin, white cell count, platelet count, urea, creatinine, albumin, lactate, C-reactive protein (CRP) and alanine transaminase (ALT), presenting diagnosis, number of organ systems affected at presentation, disposition from the ED, length of hospital stay and in-hospital mortality. The vital signs data were used to calculate the quick Sequential Organ Failure Assessment (qSOFA) score and the National Early Warning Score 2 (NEWS-2). Both qSOFA and NEWS-2 are standardised scoring tools that characterise acute illness severity, with higher scores indicating greater severity of illness and a higher risk for worse outcomes.^16,17^ The various presenting diagnoses were either microbiologically or histologically confirmed or were deemed as the most likely diagnosis based on clinical assessment, special investigations and after discussion with relevant sub-speciality clinicians. Data were thereafter exported to Microsoft^®^ Excel^®^ (Microsoft 365, Version 16.0.13029.20232) and analysed and described using either the median and standard deviation or frequency and percentages.

### Ethical consideration

Data collection commenced once ethical approval from the University of the Witwatersrand Human Research Ethics Committee (clearance certificate number: M160512) and relevant permissions were obtained. Adult patients (≥ 18 years) known to be living with HIV, including those newly diagnosed, were prospectively enrolled into the study between 07 July 2017 and 18 October 2018. As per the CMJAH ED protocol, besides patients that are already HIV-positive (either self-reported or confirmed on laboratory records of patients that previously attended the facility), all other patients attending the ED are offered HIV-rapid diagnostic testing to determine their HIV status. As per the National Department of Health (NDoH) protocol, and after obtaining consent, two different HIV-rapid diagnostic tests were performed where the HIV status was unknown. Blood was initially tested with the Abon HIV 1/2/0 Tri-line Rapid test (Abon Biopharm, Hangzhou, RR China). Reactive samples were subjected to a second confirmatory rapid test, namely, the First Response HIV 1–2.0 card (PMC Medical India Pvt, Ltd, Daman, India). Patients testing positive with both, that is, newly diagnosed as HIV-positive, were also approached for study consent and participation. For patients in whom the first test was positive but the second test was negative, whole blood was drawn and sent to the laboratory for an enzyme-linked immunosorbent assay (ELISA) HIV-test. These were only approached for study participation if the confirmatory test was positive.

## Results

During the data collection period, 29 416 patients presented to the adult medical ED triage area, of which 11 383 were triaged into the ED for further management. The remaining patients were referred to an appropriate facility in accordance with the CMJAH ED triage protocol. A total of 1308 patients were HIV-positive, of which 84 were excluded from the study as informed consent could not be obtained. A total of 1224 participants were included in the final study sample.

Table 1 describes the median (IQR) age of study participants. The median (IQR) age of the entire cohort was 36 (IQR 31–44) years, with the median (IQR) age of men being older than that of women. Other demographic characteristics, new diagnosis with HIV, ART initiation and adherence, vital signs and laboratory findings of study participants are presented in Table 2. Most participants were women (*n* = 673, 55.0%), black (*n* = 1174, 95.9%), single (*n* = 937, 76.6%) and had completed secondary school as the highest level of education (*n* = 1195, 97.6%). Those who were not South African nationals comprised a fifth (*n* = 253, 20.7%) of study participants.

**TABLE 1:** Description of the median (interquartile range) age of study participants.

Variable	Entire cohort	Male	Female
Median age (years). (IQR)	36 (31–44)	38 (32–45)	35 (30–43)

IQR, interquartile range.

**TABLE 2:** Description of demographic characteristics, human immunodeficiency virus diagnosis, antiretroviral therapy initiation and adherence, vital signs and laboratory findings of study participants.

Variable	*n*	%
**Demographic characteristics**		
Sex		
Female	673/1224	55.0
Male	551/1224	45.0
Race		
Black	1174/1224	95.9
Other†	50/1224	4.1
Marital status		
Single	937/1224	76.6
Married	287/1224	23.4
Highest level of education		
Secondary school	1195/1224	97.6
Primary school	16/1224	1.3
Tertiary education	13/1224	1.1
Nationality		
South African	971/1224	79.3
Non-South African	253/1224	20.7
**HIV diagnosis and ART initiation/adherence**		
Newly diagnosed with HIV	212/1224	17.3
ART initiated prior to ED presentation‡	761/1012	75.2
ART non-adherence	245/761	32.2
**Vital signs**		
Respiratory rate > 20 breaths/min	434/1118	38.8
Oxygen saturation < 90%	196/1117	17.5
Systolic blood pressure < 90 mmHg	116/1117	10.4
Heart rate > 110 beats/min	565/1117	50.6
**Glasgow coma scale**		
15	929/1150	80.8
12–14	176/1150	15.3
9–11	38/1150	3.3
< 9	7/1150	0.6
**Laboratory findings**		
CD_4_ < 100 cell/mm^3^	527/1105	47.6
HIV viral load > 1000 copies/mL	619/1049	59.0
**Haemoglobin**		
> 10.9 g/dL	550/1129	48.7
8–10.9 g/dL	366/1129	32.4
< 8 g/dL	213/1129	18.9
White cell count < 4.0 × 10^9^/L	170/1127	15.1
Platelet count < 150 × 10^9^/L	223/1121	19.9
Urea > 10 mmol/L	277/1069	25.9
**Creatinine**		
≤ 120 μmol/L	761/1061	71.7
121–200 μmol/L	129/1061	12.2
> 200 μmol/L	171/1061	16.1
**C-reactive protein**		
≤ 10 mg/L	164/1059	15.5
11–50 mg/L	193/1059	18.2
51–100 mg/L	186/1059	17.6
> 100 mg/L	516/1059	48.7
**Lactate**		
≤ 2.0 mmol/L	648/1118	58.0
2.1–5.0 mmol/L	387/1118	34.6
> 5.0 mmol/L	83/1118	7.4
**Albumin**		
> 34 g/L	408/1042	39.1
25–34 g/L	426/1042	40.9
< 25 g/L	208/1042	20.0
Alanine transaminase > 100 mmol/L	109/1029	10.6

Note: The denominator has been included for all variables to account for missing data.

ART, antiretroviral therapy; ED, emergency department; HIV, human immunodeficiency virus.

† , Includes Asian, Caucasian and mixed race.

‡ , Percentage calculated amongst participants who were known with HIV prior to ED presentation.

Approximately one-sixth of participants (*n* = 212, 17.3%) were newly diagnosed with HIV at presentation. Of the 1012 participants who were diagnosed with HIV prior to ED presentation, 761 (75.2%) were on ART. Of these, 245 (32.2%) were non-adherent as per the ACTG-AQ self-report questionnaire. Respiratory rate was > 20 breaths/min in 434 (38.8%) participants, oxygen saturation was < 90% in 196 (17.5%), systolic blood pressure was < 90 millimetre of mercury (mmHg) in 116 (10.4%), heart rate was > 110 beats/min in 565 (50.6%) and GCS was < 15 in 221 (19.2%) participants.

The overall median CD_4_ cell count and HIV VL were 112 (IQR, 34–295) cell/cubic millimetre (mm^3^) and 8815 (37–325 898) copies/millilitre (mL), respectively. Almost half of the study participants (*n* = 527, 47.6%) had a CD_4_ cell count of < 100 cell/mm^3^, whilst more than half (*n* = 619, 59.0%) had a VL of > 1000 copies/mL. Amongst participants on ART who reported non-adherence, the HIV VL was > 1000 copies/mL in more than two-thirds of participants (*n* = 167, 68.2%).

More than half of the participants (*n* = 579, 51.3%) presented with varying degrees of anaemia (haemoglobin < 11 grams per decilitre [g/dL]), whilst creatinine was > 120 micromole per litre (µmol/L) in 291 (23.8%), CRP was > 10 millimoles per litre (mmol/L) in 895 (74.5%), lactate was > 2 mmol/L in 470 (42.0%) and albumin was < 35 g/L in 634 (60.8%) participants.

Approximately one-fifth of participants (*n* = 244, 19.9%) presented with active tuberculosis (TB), of whom 70 (28.7%) had disseminated TB, whilst 143 (58.6%) had extrapulmonary TB (EPTB). The median CD_4_ cell count was higher, and the median HIV VL was lower amongst participants with (1) a recurrent episode of TB compared with those with a first episode, (2) TB of a single organ compared with those with disseminated TB and (3) isolated pulmonary TB (PTB) compared with those with EPTB. These and other findings pertaining to TB amongst study participants are presented in Table 3.

**TABLE 3:** Description of tuberculosis history and presentation amongst study participants.

Variable	*n*	%	CD_4_ cell count (cells/mm^3^)		HIV viral load (copies/mL)	
			Median	IQR	Median	IQR
Previous history of TB	294	24.0	63	26–176	97 948	458–657 750
TB at current presentation	244	19.9	109	37–296	1740	0–240 623
First episode of TB	216	88.5	59	25–156	106 823	599–659 109
Recurrant episode of TB	28	11.5	93	51–252	962	15–422 285
Single-organ TB	174	71.3	82	34–226	25 000	248–398 515
Disseminated miliary TB	38	15.6	37	22–87	361 172	7050–1 050 000
Disseminated non-miliary TB	32	13.1	42	14–141	157 532	2050–1 163 090
Pulmonary TB†	101	41.4	89	29–202	130 000	450–710 435
Extrapulmonary TB:	143	58.6	68	25–154	920 468	288 550–2 334 203
Miliary TB	38	15.6	37	22–87	361 172	7050–1 050 000
Pleural TB	31	12.7	139	56–313	1510	0–69 250
Abdominal TB	27	11.1	54	25–112	43 100	710–475 908
Tuberculous meningitis (TBM)	23	9.4	104	35–220	141 190	231–264 500
Tuberculous lymphadenitis	10	4.1	21	7–75	65 200	1980–184 000
Tuberculous pericarditis	9	3.7	65	47–120	3500	819–35 963
Tuberculoma	4	1.6	150	26–269	48 373	15–1 005 044
Urogenital TB	3	1.2	76	39–183	210 064	129 459–805 032
Spinal TB	2	0.8	525	512–537	2097	1918–2275
Tuberculous osteomyelitis	1	0.4	15	15–15	531 001	531 001–531 001

Note: Probable cases of tuberculosis were also included as microbiological confirmation was not available for all cases.

TB, tuberculosis; IQR, interquartile range.

† , Only includes participants with isolated pulmonary tuberculosis. The total number of pulmonary tuberculosis cases will be 171 (70.1%) if participants with both pulmonary and concurrent extrapulmonary tuberculosis are included.

Table 4 describes the most frequent presenting diagnoses amongst study participants and the corresponding median (IQR) CD_4_ cell count and HIV VL. Most participants presented with respiratory system pathology (*n* = 533, 43.5%), followed by pathology involving the genitourinary system (*n* = 249, 20.3%), gastrointestinal system (*n* = 223, 18.2%) and central nervous system (*n* = 145, 11.8%). A total of 838 (68.4%) participants presented with an infectious disease. The most common presenting diagnoses included bacterial pneumonia (*n* = 276, 22.5%), PTB (*n* = 171, 14.0%), acute gastroenteritis (*n* = 56, 4.6%), *Pneumocystis jirovecii* pneumonia (*n* = 47, 3.8%), cryptococcal meningitis (*n* = 38, 3.1%), bacterial meningitis (*n* = 30, 2.5%) and chronic gastroenteritis (*n* = 30, 2.5%).

**TABLE 4:** The most frequent presenting diagnoses and corresponding median CD_4_ cell count and human immunodeficiency virus viral load amongst study participants.

Variable	*n*	%	CD_4_ cell count (cells/mm^3^)		HIV viral load (copies/mL)	
			Median	IQR	Median	IQR
**Central nervous system**	**145**	**11.8**	**106**	**34–264**	**13 480**	**34–189 970**
Cryptococcal meningitis	39	3.2	27	12–72	130 126	1062–300 000
Bacterial meningitis	30	2.5	94	72–170	36 800	12 040–224 932
Tuberculous meningitis	23	1.9	104	35–220	141 190	231–264 500
Other	53	4.3	272	114–480	37	0–1895
**Respiratory system**	**533**	**43.5**	**71**	**13–72**	**73 300**	**2611–372 319**
Bacterial pneumonia	276	22.5	77	20–212	75 800	221–592 500
Pulmonary tuberculosis	171	14.0	89	29–202	130 000	450–710 435
*Pneumocystis jirovecii* pneumonia	47	3.8	27	18–90	429 862	98 057–551 510
Other	39	3.2	275	124–406	144	0–4670
**Cardiovascular system**	**47**	**3.8**	**232**	**84–440**	**680**	**0–20 860**
Congestive cardiac failure	18	1.5	225	114–351	100	0–308 000
Other	29	2.3	233	68–419	1870	10–8855
**Gastrointestinal system**	**223**	**18.2**	**97**	**32–259**	**1850**	**20–393 239**
Acute gastroenteritis	56	4.6	94	25–243	5500	345–874 500
Chronic gastroenteritis	30	2.5	50	11–177	72 334	389–496 905
Abdominal tuberculosis	27	3.0	54	25–112	43 100	710–475 908
Tuberculous medication-induced hepatitis	18	1.5	55	45–95	20 475	218–776 000
Other	92	7.5	208	64–400	45	0–118 750
**Genitourinary system**	**249**	**20.3**	**94**	**25–289**	**1550**	**42–230 426**
Acute kidney injury	138	11.3	57	19–162	3636	40–400 000
Chronic kidney disease	25	2.0	41	15–115	5500	582–288 500
Acute chronic kidney disease	22	1.8	67	20–220	4050	43–175 989
Urosepsis	19	1.6	300	98–643	108	31–6580
Pelvic inflammatory disease	14	1.1	298	196–421	581	0–1295
Other	31	2.5	353	188–402	498	13–48 565
**Psychiatric**	**78**	**6.4**	**320**	**110–476**	**37 450**	**217–227 750**
HIV-associated neurocognitive disorder	15	1.2	138	74–298	305 480	81 075–858 500
Parasuicide intentional overdose	14	1.1	402	300–424	10	0–3273
Schizophrenia	14	1.1	355	98–453	8360	3790–49 668
Substance-induced psychosis	13	1.1	340	162–569	48 325	252–244 394
Other	24	1.9	397	111–553	20 844	228–296 000
**Skin and soft tissue**	**49**	**4.0**	**252**	**72–417**	**39 090**	**6318–933 323**
Kaposi’s sarcoma	13	1.1	89	42–187	917 518	409 512–3 585 400
Cellulitis	8	0.7	282	243–548	2602	5–28 070
Herpes zoster	6	0.5	122	18–142	48 000	8465–816 000
Other	23	1.9	318	55–434	6050	63–272 358
**Haematological system**	**58**	**4.7**	**125**	**49–244**	**248**	**22–184 000**
Deep vein thrombosis	20	1.6	152	97–247	195	21–88 600
Lymphoma	11	0.9	79	62–127	86 727	40 218–262 547
Pulmonary embolism	10	0.8	379	168–425	155	60–734 297
Thrombotic thrombocytopenic purpura	5	0.4	48	31–199	75 500	102–624 000
Other	12	1.0	42	10–152	2260	220–213 000

IQR, interquartile range; HIV, human immunodeficiency virus.

Just over one-third presented with pathology affecting one organ system (*n* = 460, 37.6%) or two organ systems (*n* = 432, 35.2%), whilst the remainder (*n* = 332, 27.2%) had pathology affecting three or more organ systems.

Table 5 describes the qSOFA and NEWS-2 illness severity scores, patient disposition from the ED, length of hospital stay and in-hospital mortality of study participants. Of note, 196 (17.5%) had a high qSOFA score (≥ 2 points), 496 (44.4%) had a high NEWS-2 score (≥ 7 points), 813 (66.5%) required admission to the general ward and 205 (16.7%) required intensive care unit (ICU) admission. The median length of hospital stay was 4.9 (3.5–8.0) days, with approximately one-third (*n* = 394, 32.2%) requiring admission for ≥ 7 days. The overall mortality amongst study participants was 13.6% (*n* = 166).

**TABLE 5:** Quick sequential organ failure assessment and national early warning score illness severity scores, patient disposition from the emergency department, length of hospital stay and in-hospital mortality amongst study participants.

Variable	*n*	%
**qSOFA score**		
Low score (0–1 point)	921	82.5
High score (2–3 points)	196	17.5
**NEWS-2 score**		
Low score (0–4 points)	449	40.2
Medium score† (5–6 points)	171	15.4
High score (≥ 7 points)	496	44.4
**Disposition from the emergency department**		
General ward admission	813	66.5
ICU admission	205	16.7
Discharged home from ED	206	16.8
**Length of hospital stay**		
< 7 days	830	67.8
≥ 7 days	394	32.2
**In-hospital mortality**	166	13.6

ED, emergency department; qSOFA, quick sequential organ failure assessment; NEWS, National Early Warning Score; ICU, intensive care unit.

† , Includes patients with an overall low score but a score of 3 in any individual parameter.

## Discussion

To our knowledge, this is the largest single-centre study, describing the presentation of PLWH to an ED in SSA. Noteworthy findings include the large proportion of participants presenting with undiagnosed HIV, ART-treatment naivety/non-adherence, elevated HIV VL whilst on ART, other deranged laboratory parameters, HIV-related acute illness and in-hospital mortality.

During the early days of the HIV epidemic and before the introduction of highly active antiretroviral therapy, hospital admission rates amongst PLWH were substantially higher, and predominantly because of opportunistic infections and other acquired immunodeficiency syndrome (AIDS)-defining illnesses.^18^ With the widespread introduction of effective ART, the life expectancy of PLWH is approaching that of the general population, and hospital admissions in these regions have begun to reflect age-related chronic illnesses or comorbidities rather than HIV-related acute illnesses.^19^

It is well established that the early initiation of ART and efforts to optimise ART adherence have been highly effective in curtailing the transmission of HIV and reducing HIV-associated morbidity and mortality.^20,21,22,23^ Hence, it is of concern that despite the free availability of ART to all South African PLWH,^11^ over two-thirds of study participants presented with opportunistic infections and other HIV-related acute illnesses. The high percentage of participants with CD_4_ cell counts < 100 cell/mm^3^ (47.6%), HIV VL > 1000 copies/mL (59.0%) and the large number of participants newly diagnosed with HIV (17.3%), or naïve to ART (24.9%) and those non-adherent to ART (32.2%) highlight the need for an urgent public health response and the implementation of innovative strategies to improve current HIV awareness and educational programmes, as well as to increase the rates of ART initiation, ART adherence and retention in care.

A previous systematic review and meta-analysis that included 313 006 pooled adult patients from 99 studies conducted in 50 countries, with studies being mostly conducted between 2007 and 2015 and reflecting a time when access to ART had become more widespread than before this period, reported that HIV and AIDS-related illnesses (46%) and bacterial infections (31%) were the most common reasons for hospital admission in all geographical regions. Acquired immunodeficiency syndrome-related illnesses were mostly non-bacterial opportunistic infections.^24^ Comparatively, in this study, a slightly lower proportion (68.4%) of participants presented with an infectious aetiology (bacterial and non-bacterial), fewer participants were diagnosed with HIV at presentation (17.3% vs. 30%), more were on ART (75.1% vs. 43%), the median length of hospital stay was shorter (4.3 days vs. 9 days) and in-hospital mortality was lower (13.6% vs. 20%). Despite this, the median CD_4_ cell count was lower (112 cells/mm^3^ vs. 168 cells/mm^3^) in this study. Additionally, there was a higher percentage of participants in this study with bacterial pneumonia (22.5% vs. 15%) and TB (19.9% vs. 18%), whilst a lower percentage of patients were admitted with *Pneumocystis jirovecii* pneumonia (3.8% vs. 8%) and gastroenteritis (7.1% vs. 9%). Also, there were more cases of EPTB (58.6%) than isolated PTB (41.4%) in this study, in contrast to the pooled studies in which 67% presented with PTB. As the meta-analysis represented a wider demographic pool of patients, this may be a likely reason for the difference between findings of that study and this study.

A separate systematic review and meta-analysis consisting of 56 pooled studies conducted in SSA, which investigated trends in CD_4_ cell count at presentation to a medical facility between 2002 and 2013, found that the mean estimated CD_4_ cell count was 251 cells/mm^3^ in 2002 and 309 cells/mm^3^ in 2012, with no significant annual increase over the entire period. However, of the 13 studies conducted in SA, a significant increase in the CD_4_ cell count of 39.9 cells per annum (*p* = 0.02) was noted from 2002 to 2013. The overall mean CD_4_ cell count of the 13 studies conducted in SA was 257 cell/mm^3^,^25^ whereas in the current study this was lower (209 cell/mm^3^). This study is unique in that it was conducted at a tertiary-level facility, which excluded patients with low acuity conditions as they were triaged to lower-level care facilities. However, the aforementioned meta-analysis included studies conducted at ‘prevention of mother to child transmission (PMTCT) clinics’ and other lower level of care centres, which may explain the lower mean CD_4_ cell count observed in this study.

With regard to other findings of this study, anaemia in HIV-positive patients has been shown to be an independent predictor of clinical response, with a study showing that severe anaemia at baseline was associated with 13 times higher risk of death within the first year of ART initiation.^26^ Another study showed that an increasing severity of anaemia was associated with higher rates of TB and mortality, and was superior to the CD_4_ cell count as a predictive marker in patients on ART.^27^

Acute kidney injury was reported in 11.3% of study participants. Other studies reported rates of acute renal dysfunction of 2.9% – 18%,^28,29,30^ with the incidence being still high in the post-ART era.^28^ Similar to findings of this study, acute renal dysfunction has been reported more commonly in patients with a low CD_4_ cell count and high HIV VL.^29^

With regard to other significant study findings, the relatively high number of participants with an elevated CRP (84.5%), hypoalbuminemia (60.8%), hyperlactatemia (42%), thrombocytopaenia (19.9%) and a high qSOFA score (17.5%) is in line with the large number of participants presenting with severe illnesses. Previous studies have shown that elevated CRP,^31^ albuminemia,^32^ hyperlactatemia,^33^ thrombocytopaenia^34^ and higher qSOFA scores^35,36^ were predictors of mortality and poor outcomes in HIV-positive individuals.

## Limitations

Limitations of this study are that this was a single-centre study, and that data were collected over a relatively short duration of 15 months. Also, with regard to the median CD_4_ cell count and HIV VL values described in Tables 3 and 4, we did not account for differences between participants who were on ART and those who were not on ART or were ART non-adherent. Furthermore, as the study was conducted at a tertiary-level academic hospital and excluded patients with less severe presenting illnesses, our cumulative findings are likely to be an overestimate and not be fully reflective of the wider HIV-positive population residing within the drainage area of the hospital. A further limitation is the lack of data on chronic comorbid diseases, such as hypertension and diabetes, and the absence of follow-up outcomes post-discharge.

## Conclusion

Despite the passage of more than 30 years of the HIV pandemic in Africa, PLWH are still at risk of serious morbidity and inappropriate mortality. In order to achieve the target of ending HIV by 2030 in SA, a more urgent public health response is required. This must include more innovative strategies to improve HIV awareness, new thoughts with regard to prevention, upgrading of ART services and dedication to the retention of all PLWH in care.
